# The Prevalence of Liver Cytolysis in Children with *Helicobacter pylori* Infection

**DOI:** 10.3390/children9101498

**Published:** 2022-09-30

**Authors:** Ancuta Lupu, Ingrith Crenguta Miron, Anca Lavinia Cianga, Andrei Tudor Cernomaz, Vasile Valeriu Lupu, Cristina Gavrilovici, Iuliana Magdalena Stârcea, Elena Tarca, Dragos Catalin Ghica, Silvia Fotea

**Affiliations:** 1 Pediatrics Department, “Grigore T. Popa” University of Medicine and Pharmacy, 700115 Iasi, Romania; 2 III-rd Medical Department, “Grigore T. Popa” University of Medicine and Pharmacy, 700115 Iasi, Romania; 3 Department of Pediatric Surgery, “Grigore T. Popa” University of Medicine and Pharmacy, 700115 Iasi, Romania; 4 Preventive Medicine, “Grigore T. Popa” University of Medicine and Pharmacy, 700115 Iasi, Romania; 5 Pediatrics Department, Faculty of Medicine and Pharmacy, “Dunarea de Jos” University of Galati, 800008 Galati, Romania

**Keywords:** liver cytolysis, *Helicobacter pylori*, children, transaminases

## Abstract

(1) Background: The relationship between *Helicobacter pylori* (*H. pylori*) infection and liver disease has been discussed for many years, but the association between the infection and liver cytolysis in children has been insufficiently explored. In our study, we evaluate this relationship in a pediatric population from the northeast of Romania. (2) Methods: A retrospective study of children with *H. pylori* infection and liver cytolysis was conducted on a group of 1757 children, admitted to a pediatric gastroenterology regional center in northeast Romania over 3 years. (3) Results: Liver cytolysis syndrome was present in 112 children of both sexes. Of the 112 children, 20 children (17.9%) also had *H. pylori* infection. In the statistical analysis, we noted a significant association between liver cytolysis syndrome and *H. pylori* infection (χ^2^; *p* < 0.001). (4) Conclusions: This relationship requires further in-depth studies that also consider certain parameters that may influence the results of these correlations. In addition, we point out the need for further analyses evaluating, in terms of the histopathological changes in each liver disease, the efficacy of *H. pylori* eradication.

## 1. Introduction

*Helicobacter pylori (H. pylori)* is a spiral bacterium that is Gram-negative, with increased motility determined by the presence of multiple unipolar flagella. It is microaerophilic and produces urease [1]. *H. pylori* populates the mucus adjacent to the gastric mucosa. The bacterium’s shape, motility, reduced oxygen requirement, adhesion molecules that are trophic to certain gastric cells, and urease production are all important adaptive characteristics that help the body survive in an acidic environment. Bacterial urease converts urea to ammonium and bicarbonate, neutralizing gastric acid and providing protection in a hostile environment, strong stomach acid [2,3]. It is usually transmitted in childhood by person-to-person interaction from saliva and it leads to many diseases, such as chronic gastritis, gastric mucosa-associated lymphoid tissue (MALT) lymphoma, peptic ulcer disease and gastric cancer [4]. The Correa theory states that *H. pylori* infection determines sequential phenomena, starting from chronic gastritis and continuing to jejunal metaplasia and dysplasia, and in the end, to gastric cancer [5].

The importance of *Helicobacter pylori* infection in the mechanisms of extra-gastric injury along with its impact on the pathogenesis of cardiovascular and metabolic diseases have been vastly explored. Moreover, there is evidence that *H. pylori* is also involved in liver disease, being a key factor in the evolution of non-alcoholic fatty liver disease, insulin resistance, liver fibrosis, non-alcoholic steatohepatitis and cirrhosis [6]. Plus, during *H. pylori* infection, we can expect a worsening of liver inflammation along with autoimmune manifestations that involve both the liver and the biliary tract [7]. One of the possible mechanisms that can lead to liver fibrosis and possibly cancer in vivo is stimulation of the podosome formation and collagen accumulation in the hepatocytes, which was shown by Le Roux-Goglin et al. in cultured mouse hepatocytes samples [8]. An important meta-analysis described a greater prevalence in developing countries (Latin America, Asia) than in developed ones (Europe, the United States) [9].

The epidemiological data on *H. pylori* infection in Romania among the pediatric population show that there has been a decrease in the prevalence of *H. pylori* infection, a situation that may be correlated with better socioeconomic conditions [10].

However, Yuan et al. indicated in their review that *H. pylori* infection still presents a high prevalence among the pediatric population all over the world, signaling the importance of the infection [11].

Even though the information regarding the relationship between *H. pylori* infection and liver cytolysis has been explored for a long time, we present novel results from the northeast region of Romania on a pediatric population, focusing on the prevalence of the hepatic modifications.

## 2. Materials and Methods

A retrospective study was conducted on 1757 children, admitted to a pediatric gastroenterology service in Northeast Romania over 3 years, looking for *H. pylori* infection and liver cytolysis.

Patients treated previously to eradicate *H. pylori*, those who had undergone previous treatment with antibiotics or acetaminophen, patients with endoscopic evidence of active gastrointestinal bleeding, esophageal stricture or esophagitis secondary to systemic diseases and patients with any history of gastric or esophageal surgery, viral hepatitis, bile duct obstructions, drug-induced hepatitis, cirrhosis or hepatocellular carcinoma were excluded [12].

According to the existing data in the literature on the relationship of *H. pylori* infection in children with hepatic cytolysis, we evaluated the values and variations of the serum levels of liver enzymes (alanine transaminase (ALT) and aspartate transaminase (AST)).

Upper gastrointestinal endoscopic examinations were performed on all patients under intravenous sedation using Pentax and Olympus video pediatric gastroduodenoscopes to identify evidence of macroscopic abnormalities. In children aged below 10 years, general anesthesia was used. Biopsies were prevalently from the gastric corpus and antrum, which were collected during endoscopy for rapid urease testing and for histological and bacteriological examination [12].

### 2.1. Ethical Considerations

Informed consent was obtained from all patients/caregivers, and the “St. Mary” Children Emergency Hospital Ethics Committee’s approval number 31490 from 29 October 2021 was obtained for this study to be published.

### 2.2. Statistical Analysis

The research used data from patient observation charts, discharge papers from the hospital database and endoscopy results.

The data were analyzed using the IBM SPSS 17.0 platform and the Microsoft Excel software.

Non-parametric chi-squared tests were utilized to highlight differences in parameters amongst the individuals included in the study.

The obtained results should be extrapolated with caution given that the data came from the analysis of a sample of a certain size and influenced by a number of factors, such as seasonality and accessibility to healthcare.

## 3. Results

Among the 1757 valid patients, there were 542 with *H. pylori* infection; the study group structure is reported in Table 1.

There were significant differences between gender-stratified subgroups in terms of *H. pylori* infection and the presence of liver cytolysis (Table 2).

The presence of liver cytolysis seemed linked to the presence of *H. pylori* infection (Table 3).

Given these preliminary data, we focused on the 542 *H. pylori*-positive cases; females represented more than two-thirds of the sample with a percentage of 73.2% (397 girls), compared to less than one-third represented by males with a percentage of 26.8% (145 boys) (Table 4).

The distribution of cases according to the area of origin showed a higher frequency of children from rural areas with a percentage of 75.3% (408 children), compared to those from urban areas with a percentage of 24.7% (134 children), out of the total number of cases with *H. pylori* infection. (Table 4)

Of the 542 cases of *H. pylori*-infected patients, hepatocytolysis was found in 20 children (3.7%); there were significant differences between genders and in terms of the mean age (Table 5).

The statistical analysis showed a significant association between *H. pylori* infection and liver cytolysis syndrome (χ^2^; *p* < 0.001).

The calculation of the parameters of chance (OR = 0.51) and risk (RR = 0.56) did not bring up additional parameters, with the estimated value being insignificant.

The statistical analysis also demonstrated that the prevalence of liver cytolysis was slightly higher among the female population with H. *pylori* infection, with 11 cases (54.9%), compared to the male population, representing 45.1% (9 cases).

**Figure 1:**
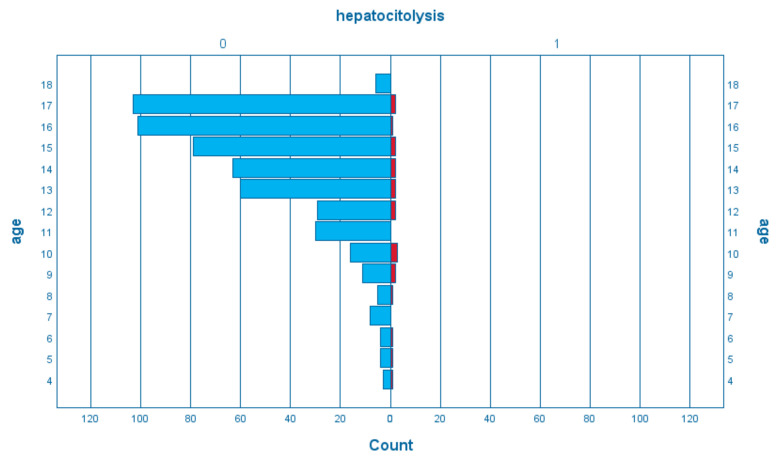
The structure of the group of patients according to age and the presence of hepatocytolysis (blue—absent, red—present).

When analyzing the means of the group of liver cytolysis cases and the age group data (Figure 1), we observed a younger average age of the patients with increased transaminases (11.45 + 3.859) compared to the children who did not present hepatocytolysis (14.20 + 2.741), the average difference being 2.75 years (95% confidence interval (−3.99)–(−1.49), *p* < 0.05).

## 4. Discussion

It seems that *Helicobacter* spp. is involved in the pathogenesis of chronic liver disease and liver carcinoma since it has been isolated from liver samples from a variety of mammals [13,14]. However, the direct influence of *H. pylori* on liver diseases remains unknown.

In humans, an *H. pylori*-like microorganism was first detected in the resected gallbladder mucosa of a gallbladder patient [15]. The authors of various studies have reported the presence of *H. pylori* in liver samples from patients with primary sclerosing cholangitis and primary biliary cirrhosis by molecular biology techniques [16,17].

In a study conducted on 174 patients by Dogan et al., a relationship between fatty liver and *H. pylori*-positive patients was described, along with an enlargement of the liver and the spleen with ultrasonography [18].

A few authors have also reported a very high prevalence of anti-*H. pylori* antibodies in patients with cirrhosis compared to the control group [19]. Although *Helicobacter* spp. DNA was found in liver samples of individuals with primary liver carcinoma, the question remained whether these findings mean real liver colonization or whether *H. pylori* DNA was because of the retrograde transfer of DNA from the duodenum to the liver. Isolation of *H. pylori* on culture medium by means of liver samples provided proof of real bacterial colonization [17,20].

Furthermore, it is known that hepatitis B virus (HBV) and hepatitis C virus (HCV) infections represent some of the common causes of liver cirrhosis. It was already postulated that *H. pylori* infection is associated with HBV- and HCV-related cirrhosis. Moreover, *H. pylori* infection is more common in cirrhotic individuals with hepatic encephalopathy than in those who do not have it [21].

It was further postulated that *H. pylori* infection may promote non-alcoholic fatty liver disease (NAFLD) through the impact of possible inflammatory stimuli from Gram-negative microaerophilic *H. pylori* on the portal circulation and via the increase in chronic inflammation, systemic insulin resistance and altered adipocytokine secretion patterns [22,23]. The same relationship was present in important studies conducted by Chen et al. in China [24].

On the other hand, while Yan in Bali found that the infection of *H. pylori* represents an independent risk factor for NAFLD [25], Wang et al. observed in an impressive study on 71,633 participants in China that the same infection was not a risk factor for liver function damage in patients with NAFLD (AST, *p* = 0.911; ALT, *p* = 0.237) [26].

Although we cannot demonstrate the definite involvement of *H. pylori* in hepatic pathology in children, nevertheless, in our study, the statistical analysis showed a strong significant relationship between *H. pylori* infection and liver cytolysis syndrome (χ^2^; *p* < 0.001). Liver cytolysis syndrome was present in 112 children of both sexes from the initial batch of 1757 patients. Of the 542 children with *H. pylori* infection, 20 children had liver cytolysis syndrome.

However, our results are not consistent with the data extracted from a cross-sectional study conducted on 630 patients by Galal et al., who did not find any correlation between the presence of *H. pylori* infection and the elevation of liver enzymes (*p* = 0.93 for ALT and *p* = 0.86 for AST) [27].

It is also necessary to consider other pathologies that cause the increase in liver enzymes (other infections, diseases of nutrition and metabolism, endocrine diseases, autoimmune diseases, intestinal parasitosis). However, the increase in transaminase values was mild (below 3 × he normal values).

In 2014, Salehi et al. observed that out of a total number of 107 patients with liver cytolysis included in their study conducted in Iran, 93 of them presented an important decrease in the serum levels of transaminases after having completed the treatment for a concomitant *H. pylori* infection, suggesting a potential relationship between *H. pylori* infection and alteration of liver function test results [28].

The same result was obtained by Maharshi et al. in their study conducted on 80 patients for whom reduced hepatic steatosis and liver enzymes were observed after eradicating the *H. pylori* infection [29].

These findings do not correlate, though, with the results of Graham et al., who did not obtain a change in the values of liver enzymes after *H. pylori* infection treatment, suggesting the potential presence of an extra-hepatic source of liver cytolysis or a simultaneous host genetic susceptibility to *H. pylori* infection and hypertransaminasemia [30].

Săsăran et al. stated that *Helicobacter pylori* infection is still presenting a public health problem in Romania, particularly in children from rural areas. Our findings are consistent with these results, showing a relationship between a rural environment and children with *H. pylori* infection and liver cytolysis syndrome [31].

Beyond this, in their impressive review, Park et al. reiterated the fact that age and sex cannot be correlated with *H. pylori* infection in children, and our study proves the same for the age variable [32]. On the other hand, for the gender variable, we encountered a higher prevalence of female patients with *H. pylori* infection (397 cases) compared to male patients (145 cases).

As for liver cytolysis, unlike the results from a very complex retrospective, observational and descriptive study conducted in Spain where a predominance of the male sex was noted, in our study, we obtained similar results for the gender variables among the patients with liver cytolysis, with 11 females and 9 males [33].

Although facts regarding the associations between *H. pylori* and liver disease pathogenesis are gathering, the existing data do not lead us to an unequivocal conclusion. The need to establish a definite association between hypertransaminasemia, which presents a cause of concern among the pediatric population, and infection with *H. pylori* remains of great interest, especially considering the high prevalence of *H. pylori* infection in the general population.

## 5. Conclusions

Concerning extra-gastric manifestations of *H. pylori* infection, the present statistical analysis showed an association between *H. pylori* infection and liver cytolysis syndrome, but this relationship requires further in-depth studies that also consider certain parameters that may influence the results of these correlations.

We also point out the need for further analyses evaluating, in terms of histopathological changes in each liver disease, the efficacy of *H. pylori* eradication, the long-term clinical effects of such a potential relationship and the evaluation of multiple *Helicobacter pylori* strains in hepatic diseases among the pediatric population.

## Figures and Tables

**Table 1:** Study group with/without *H. pylori* infection.

Gender	Number of Cases	Percentage
female	1210	68.9
male	547	31.1
**Living conditions**		
rural	1114	63.4
urban	643	36.6
**Liver cytolysis**		
absent	1645	93.6
present	112	6.4
***Helicobacter pylori* infection**		
absent	1215	69.2
present	542	30.8

**Table 2:** Differences between gender-stratified subgroups.

	*Helicobacter pylori* Infection		Liver Cytolysis	
	not present	present	not present	present
Gender				
female	813 (66.9%)	397 (73.2%)	1154 (70.1%)	56 (50%)
male	402 (33.1%)	145 (26.8%)	491 (29.9%)	56 (50%)
	*p* = 0.005 chi-squared		*p* < 0.0001 chi-squared	

**Table 3:** *H. pylori* and liver cytolysis.

	Liver Cytolysis	
*Helicobacter pylori*	present	not present
present	20 (17.2%)	522 (31.7%)
not present	92 (82.1%)	1123 (68.3%)
	*p* = 0.001 chi-squared	

**Table 4:** Structure of the group of patients with *H. pylori* infection.

Gender	Number of Cases	Percentage
female	397	73.20
male	145	26.80
Living conditions		
rural	408	75.30
urban	134	24.70
Liver cytolysis		
absent	522	96.30
present	20	3.70

**Table 5:** *Helicobacter pylori*-positive study group structure in relation to hepatocytolysis.

	*Helicobacter pylori*-Positive	Liver Cytolysis		
	total	not present	present	
Gender				
female	397 (73.2%)	386 (73.9%)	11 (55%)	*p* = 0.05 chi-squared
male	145 (26.8%)	136 (26.1%)	9 (45%)	
Age (years)	14.1 +/− 2.8	14.2 +/− 2.7	11.45 +/− 3.8	*p* = 0.005 Student’s *t*-test mean difference 2.7 95% confidence interval 0.9–4.5

## Data Availability

The data presented in this study are available on request from the corresponding author.
